# Access to Healthcare and Social Protection among Migrant Workers in Thailand before and during COVID-19 Era: A Qualitative Study

**DOI:** 10.3390/ijerph19053083

**Published:** 2022-03-06

**Authors:** Watinee Kunpeuk, Sataporn Julchoo, Mathudara Phaiyarom, Pigunkaew Sinam, Nareerut Pudpong, Tharani Loganathan, Huso Yi, Rapeepong Suphanchaimat

**Affiliations:** 1 International Health Policy Program, Ministry of Public Health, Tiwanon Road, Nonthaburi 11000, Thailand; sataporn@ihpp.thaigov.net (S.J.); mathudara@ihpp.thaigov.net (M.P.); pigunkaew@ihpp.thaigov.net (P.S.); nareerut@ihpp.thaigov.net (N.P.); rapeepong@ihpp.thaigov.net (R.S.); 2 Educational Service Unit, Sirindron College of Public Health, Chonburi 20000, Thailand; 3 Centre for Epidemiology and Evidence-Based Practice, Department of Social and Preventive Medicine, University of Malaya, Kuala Lumpur 50603, Malaysia; drtharani@ummc.edu.my; 4 Saw Swee Hock School of Public Health, National University of Singapore and National University Health System, Singapore 117549, Singapore; ephyh@nus.edu.sg; 5 Division of Epidemiology, Department of Disease Control, Ministry of Public Health, Nonthaburi 11000, Thailand

**Keywords:** migrant, health policy, social protection, COVID-19

## Abstract

Thailand is a popular host nation for international migrant workers, particularly those from Cambodia, Lao PDR, and Myanmar. Thailand has introduced approaches to protect their rights for health and social welfare, using various mechanisms over many years. However, the implementation of these policies is dynamic and has been influenced by national security, economic necessity, and public health concerns. The aim of this study was to explore how Thailand designs and implements health and social welfare policies for migrants in Thailand, both before and during COVID-19. A qualitative analysis was used alongside interviews with 18 key informants in various sectors in this field. Thematic coding was applied. Results show that there were seven key themes emerging from the analysis, including: (i) sustainability of the HICS; (ii) people dropping out from the Social Security Scheme (SSS); (iii) quality of health screening in the Memorandum of Understanding (MOU) migrants; (iv) health screening problems and state quarantine management in response to COVID-19; (v) managing the migration quota and dependency on migrant workers; (vi) influx of migrants in the backdrop of COVID-19; and (vii) poor living conditions of migrants and the impact of COVID-19. The majority of interviewees agreed that undocumented migrants is a critical concern that impedes access to migrants’ health and social welfare. This situation was especially pronounced during the second wave of COVID-19 in Thailand, which took hold in migrant communities. In the short term, the poor living conditions of migrants urgently need to be addressed in order to contain and mitigate this crisis. In the long term, there needs to be an improved health system design that includes migrants, regardless of their immigration status. This requires intersectoral policy coherence, including the hastening of nationality verification to sustainably mitigate undocumented migrants.

## 1. Introduction

Southeast Asia is one of the world’s most dynamic regions, with a substantial volume of migrant workers moving within the region, as well as between the region and the rest of the world [1]. Destination countries are often those with declining birth rates and a high demand for industrial sector labour, such as Brunei, Malaysia, Singapore, and Thailand. Migration in the region became more pronounced after the ten member states of the Association of Southeast Asia Nations (ASEAN) agreed to work towards full economic integration in the so-called ASEAN Community by 2015 [2].

Globally, it is estimated there are over 20 million migrants from ASEAN member states, about seven million of whom have migrated within the region [3]. By using the “push and pull” theory based on neoclassical equilibrium models [4,5,6], evidence suggests that poverty and indebtedness are key factors that aggravate both emigration and immigration in ASEAN member states. Higher wages in some nations were a significant pull factor that attracted migrant workers from others [7]. In contrast, according to the historical-structural paradigm or conflict theory, the role of businesses in the state is amongst the major causes of labour migrants and migration. It comes in the forms of both forced and voluntary migration to provide a cheap labour force to maintain the economy of a more affluent nation. This phenomenon is likely to create inequitable distribution of economic and political powers across the member states [5,6,8].

Net-sending or net-receiving countries are identified according to migrant profiling. Cambodia, Indonesia, Lao PDR, Myanmar, the Philippines, and Vietnam are net-sending countries, while Brunei, Malaysia, Singapore, and Thailand are net-receiving countries of labour migration [9]. While intra-ASEAN migration has grown enormously over the last two decades, most of it is concentrated in just a few corridors, reflecting a critical imbalance of flows. Over 90% of the seven million intra-ASEAN migrants in 2015 moved to just three countries: Malaysia, Singapore, and Thailand [10].

Of course, all these migrants have healthcare needs to varying extents. A large amount of literature explores the health system function of providing care for migrants from the perspectives of receiving countries. For example, Guinto et al. [1] investigated how certain member states of ASEAN implemented health services for migrants. Suphanchaimat et al. [3] described the chronological evolution and challenges of public insurance schemes for migrants in Thailand. Loganathan et al. [11] examined the interaction between government-mandated insurance schemes and the healthcare access of migrants in Malaysia. However, evidence that sheds light on the gaps between policy intention and policy implementation regarding health and labour protection and health education promotion is quite sparse.

In addition, the unforeseen arrival of the coronavirus disease 2019 (COVID-19) has left migrant workers especially vulnerable and susceptible to the crisis [12]. They are prone to face additional stresses due to poor living conditions, and the implementation of protective measures such as travel restrictions, physical distancing, and personal hygiene and safety can be impossible to adhere to [12,13]. Moreover, they face greater barriers to healthcare access and are likely to excluded from the healthcare system in host nations [12,14]. As such, dedicated support for these populations is required to ensure they will not be neglected by national public health systems, and that health equity for diverse cultural communities is promoted [12,15].

Thailand is the most significant host among ASEAN members, being the prime destination for many migrant workers over many years. Most of the inflow migrants are from neighbouring countries, namely, Cambodia, Lao PDR, and Myanmar (CLM) [16]. In 2018, an estimated three to four million migrants were living in Thailand, with about one and a half million living with precarious status (undocumented migrants). Some did not hold a valid passport [17]. It was estimated in February 2021 that the number of undocumented migrants requesting to be registered was approximately 0.7 million [18]. However, the Thai government established the One Stop Service (OSS) as an approach to mitigate the problems of undocumented migrants. The mechanisms of the OSS include collecting personal data records; undertaking health examinations for undocumented migrants in collaboration with the Ministry of Public Health of Thailand (MOPH); granting residence permits under the approval of the Ministry of Interior (MOI); and cooperating with the Ministry of Labor (MOL) for work permit issuance. Another approach is to import migrant workers with a legal mechanism called the bilateral Memorandum of Understanding (MOU). Both MOU and OSS migrants need to pass a health screening for serious communicable diseases, such as tuberculosis, filariasis, and elephantitis, before being issued a work permit [19]. The majority of MOU migrants undertake the health check in Thailand in private hospitals (with certified standards as approved by the Hospital Accreditation System, or the Joint Commission International or International Standardization Organisation) [20].

In Thailand, financial protection for migrant workers is separate from that of the Thai population. Due to the successful implementation of universal health coverage (UHC), in 2015, 98.5% of the Thai population was insured by three public health insurance schemes, including the Universal Coverage Scheme (UCS), the Civil Servant Benefit Scheme (CSMBS), and the Social Security Scheme (SSS) [21]. However, non-Thai populations working in the informal sector are insured through a different system to mainstream public health insurance. Key mechanisms of migrant health insurance include: (i) the SSS covering migrant workers in the formal sector; and (ii) the Health Insurance Card Scheme (HICS) covering migrant workers from Cambodia, Lao, Myanmar, and Vietnam in the informal sector [3]. The SSS was established under the Social Security Act 1990, 1994, 1999, and the Workmen’s Compensation Act 1994 [22]. The SSS insures migrant workers in the formal sector regardless of their nationality. It is compulsorily run by a payroll tax with tripartite contributions (migrant workers, employers, and the government). Migrant workers need to pay 5% of their income to the SSS fund, with 5% payment by employers and 2.75% payment by the government [22]. The SSS includes the following benefits: medical care, unemployment, stipend, retirement fund, occupational injury compensation, and delivery compensation for mothers [22]. Migrant workers in the informal sector have the right to be insured by the Health Insurance Card Scheme (HICS), which is regulated by the Division of Health Economics and Health Security (DHEHS) at the MOPH. Unlike the SSS, the HICS benefit covers only the cost of outpatient and inpatient care, health promotion, and disease prevention services; it does not provide other benefits [19]. Applicants of the HICS need to pay THB 3800 (USD 119) for two-year coverage.

Due to the fact that Thailand shares a very long border with its neighbouring countries, the influx of undocumented migrants still continues [3]. Although the government attempts to address this problem by various means (including the OSS and the MOU), it seems that the nationality verification (NV) process still faces many challenges, and the volume of undocumented migrants is believed to be underestimated [20]. Since 2020, COVID-19 and its impact have put pressure on the global economy. In Thailand, migrant communities have been affected by COVID-19 infections; they are overwhelmed by poor living conditions, overcrowding, a lack of hygienic supplies, and insufficient knowledge to contain such pandemic [23]. Despite restrictions on travel and relocation during the crisis, the Thai economy still relies on imported migrant workers. In July 2020, the MOL announced that it would recruit new MOU migrants with strict health prevention measures [24]. They were required to have a negative health screening result and quarantine for 14 days. However, about 82% of employers did not agree with the criteria as they needed to bear this expense themselves and asked the government to share some of the cost. In June 2021, the infection rate reached a critical period, and then the Thai government imposed far stricter regulations to curb this rapid pandemic trend. The new measures were implemented, including: a ban on restaurant dine-ins; closure on shopping malls; prohibition on a gathering of more than 20 people; and the shutdown of construction sites and workers’ camps. These measures were in place in Bangkok, its surrounding provinces, and in three provinces in the southern region [25]. It is undeniable that migrant workers who were key part of construction industry were affected by the order. In July 2021, the MOL launched a document to terminate a COVID-19 screening test in documented migrant workers who were at risk [26]. Therefore, such a document created unambiguity in the implementation of migrant policy and government support for those groups during the crisis situation.

Therefore, a better understanding on the policy design to facilitate access to healthcare and address the challenges in utilizing health services among cross-border migrants is imperative, and Thailand can serve as a good case study. We hope that this study will provide insights to improve policy coherence and shared responsibility among member states in the ASEAN. The objective of this study was to investigate how Thailand designs and implements policies to protect the health of migrants living on their soil during COVID-19. The challenges of care faced by migrant workers against the backdrop of COVID-19 were also examined. Note that the concept of health encompasses many aspects of wellbeing, not just physical health.

## 2. Materials and Methods

### 2.1. Study Design and Conceptual Framework

**Figure 1:**
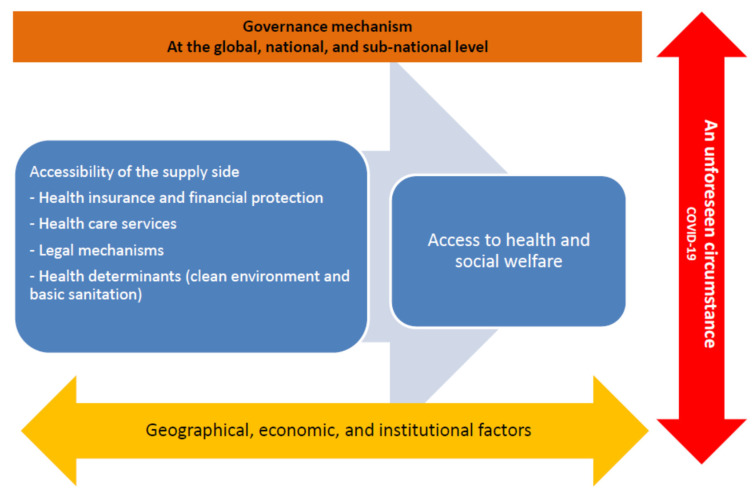
A health systems framework for migrants’ access to health and social protection (adapted from Abubakar et al. [27] and Levesque et al. [28]).

A qualitative cross-sectional study was employed. A health systems framework, adapted from Abubakar et al. [27] and Levesque et al. [28], for migrants’ access to health and social protection was used as a guideline for constructing the question guides for the analysis (see Figure 1). This framework is based on the concept of health equity, considering the dimensions of human rights and equal access to healthcare. At the macro level, it is influenced by geographical, economic, and institutional factors. Additionally, governance mechanisms can shape protections in the country. However, in this study, the focus was mainly on the supply side from the perspective of the providers as there were challenges in collecting data from migrant workers, who were key demand-side informants during the COVID-19 crisis. Moreover, the framework was applied by accounting for the effect of COVID-19 on the health system factors.

### 2.2. Samples and Data Collection

A purposive sampling method was used to target interviewees who had experience working to support migrants’ access to healthcare. Experts in the field of migrant-related policies suggested potential professionals in different governmental and nongovernmental agencies. The target migrant population, as the focus of the interview, was low-skilled migrant workers from neighbouring countries including CLM nations as these migrant workers are considered to be the majority of the overall migrant worker population in Thailand and have been the main target of migrant policies of the government [16]. Cross-border migrants with either legal or illegal status were excluded. The term “cross-border migrants” refers to “any person who is outside a State of which he or she is a citizen, national, place of birth, or habitual residence” as mentioned by the Office of the High Commissioner for Human Rights (UN Human Rights) (OHCHR) [29]. According to this reference, the meaning of cross-border migrants is defined broadly. Rather, in this study, the term “migrant workers”, who were the focus of this study, were applied. This population refers to migrants who are looking for job opportunities or have jobs in Thailand. However, high-skilled professionals, such as expatriates, tourists, asylum seekers and refugees, diplomats, and long-stay migrants were not included.

A total of 18 key participants were included in the first phase of the interview, including those from the health sector, nongovernmental organisations, international organisations, and academic institutions (see Table 1). All of them were primarily contacted by phone, and then face-to-face interviews were undertaken. The interviewees included five participants from the MOPH; two from the health sector beyond the government; two from governmental agencies; seven from non-governmental organisations (NGOs); and two from the academic sector. The status of participants ranged from policymakers to migrant health partners working in local areas. The majority of participants had worked in the field of migrant health policy for more than 10 years in senior positions. Snowball sampling was carried out if interviewees suggested further participants. Due to travel restrictions during COVID-19, a phone interview was undertaken with six participants. The interview length was approximately an hour. Interview topics had a broad scope around five issues, including: (i) interviewees’ experiences with migrant health policy at any level; (ii) viewpoints towards national migrant policy and their dependents related to health and social welfare; (iii) the impact of COVID-19 on migrant policy in relation to health and social welfare and preparedness of national support for migrants and their dependents; (iv) recommendations for the implementation of migrant policy at the national and international levels; and (v) national insecurity affected by political movement in neighbouring countries, especially Myanmar.

### 2.3. Data Analysis

Inductive and deductive thematic coding was applied in the data analysis. The interviews were audiotaped and transcribed verbatim and then manually coded in an Excel programme for thematic analysis. The themes were broadly classified into four areas. These included health insurance and financial protection for migrant workers; healthcare services; factors and challenges affecting health services; and legal mechanisms to protect the migrants’ right to health. However, for the health determinants of migrants, the focus was on clean environments, living conditions, and basic sanitation as part of occupational health and safety, which are significantly linked to the spread of COVID-19. After that, open coding was performed based on inductive thematic analysis, and the findings were categorised into subthemes emerging from the deductive coding. The interviewees were coded as letters to maintain anonymity. Document reviews on grey literature and online documents were undertaken to explore more relevant information, such as updates on health insurance arrangements, the impact of COVID-19 on migrants’ health and wellbeing, and the recent notifications of the MOL and MOI. This approach was part of triangulation to check the validity of the study results.

### 2.4. Ethics Consideration

Ethics approval was obtained from the Institute for the Development of Human Research Protections in Thailand (IHRP 340/2562). All participants were asked to sign a consent form on a voluntary basis. The interviewees were free to stop the interview at any time if they felt uncomfortable or unable to participate in the discussion. Individual identification was kept anonymous.

## 3. Results

Seven key themes emerged from the analysis. The key themes included: (i) sustainability of the HICS; (ii) people dropping out from the SSS; (iii) quality of health screening in MOU migrants; (iv) health screening problems and state quarantine management in response to COVID-19; (v) managing the migration quota and dependency on migrant workers; (vi) influx of migrants in the backdrop of COVID-19; and (vii) poor living conditions of migrants and the impact of COVID-19. Key findings for each subtheme are described as follows.

### 3.1. Sustainability of the HICS

Some interviewees suggested that the concept of the HICS itself might contradict fund management by using the provider–purchaser split, as the MOPH acts as the insurer and the provider at the same time. It is obvious that there are different structural mechanisms in the SSS, which is run by the MOL, in comparison to the HICS, which is managed by the MOPH. Some interviewees suggested that for the HICS, an independent body could be established to manage the HICS funding separately in order to avoid a conflict of interest (COI). However, currently, the MOPH acts as a provider of health services and runs the health system supporting migrants. At the same time, it is also a manager in the operation of the HICS funding.

“*I think there should be an independent body to manage the HICS funding which is not the MOPH. We should separate a provider from the funding manager (to prevent COI). The MOPH can be a provider to manage health care services and the health systems overall.*”

—Public Health Officer PA-5

A concern about the smaller pooled funding of the HICS was raised (the number of the HICS beneficiaries is about one million). One participant mentioned the sustainability of the HICS funding, where other beneficiaries of highly skilled workers might be included in the scheme in order to have greater pooled funding at the central authority. In comparison with private health insurance, the MOPH can communicate advantages of the HICS benefits to these beneficiaries, including better health promotion and chronic disease packages.

“*As the HICS funding is not that big, we can include more beneficiaries of high-skilled workers to maintain the sustainability of this funding pool. We can also inform them about advantages of HICS packages that cover health promotion and non-communicable diseases in order to compete with private health insurance.*”

—NGO PA-2

In addition, some participants mentioned the idea of integrating the HICS (for migrants) with the UCS (for Thais). Some participants referred to the United Kingdom (UK) health insurance scheme and the National Health Service, which is the only organisation responsible for all beneficiaries or ordinary residents in the UK [30]. Ordinary residents can include legal immigrants if they stay in the UK for longer than three years. From this idea, inclusiveness in the healthcare system can be promoted by insuring all people, including Thai and non-Thai populations under the same healthcare insurance scheme with equal access. At the same time, it can reduce the operational cost of health insurance funding. However, it is not practical at present as under the National Health Security Act in 2002 only citizens with Thai nationality have the right to UHC [31].

“*To set up on a single health insurance system like the UK can be a way to improve health and social equity. For the operational cost, I think this approach could save more money too as the management of different health insurance schemes will become less complex. However, it can be difficult in practice because the UHC law includes only beneficiaries with Thai nationality only.*”

—Public Health Officer PA-5

### 3.2. People Dropping out of the SSS

All employees who are working in the formal sector regardless of their nationality are insured by the SSS. The SSS benefits cover nine areas of health and social welfare. These include medical care, sickness, unemployment, old age, employment injury, family maternity, invalidity, and survivors’ benefit [20,22]. However, in practice, employers and migrant employees have shown reluctance to make a payroll contribution to the SSS because they considered it as a financial burden, even though the SSS is legally binding [20]. During the interview, a concern was raised about whether employers may hesitate to subsidise the SSS fund if their migrant workers have only worked for them for only a short period of time. Even though there are many SSS fringe benefits, some benefits are underutilised [32]. SSS benefits such as unemployment and survivors’ benefits were less utilised by migrant workers, especially for those who stayed in Thailand for just a short period of time. At the same time, some employers raised concerns about cost of the insurance; they did not want to be part of the payroll contribution for the SSS fund if they foresaw that some migrant employees would stay in Thailand for few years. Moreover, a public health officer believed that some migrant workers had limited information about the SSS benefits, which also contributed to low healthcare utilisation. Therefore, he suggested that a migrant information system for the SSS needed to be in place to track and report up-to-date payments.

“*Some migrant workers stay in Thailand for a short period of time, and some of them often change their employers. So migrants’ employers would not be satisfied once their migrant workers move away and they do not want to pay for the SSS fund because somehow it is not cost effective (even it is legally binding).*”

—Public Health Officer PA-5

“*There is inequity of access to the SSS information between migrant workers and the Thais. Some migrants they do not know once their payment will be sent to the SSS or not. And it is too late that they can use this SSS when they get sick and want to do medical reimbursement. So I would like to see the proper SSS information system to help them track their payment and know their right.*”

—NGO PA-7

During the COVID-19 crisis, the MOL announced a financial relief for migrant workers who were forced into unemployment. However, the criteria for receiving this benefit were quite strict: (i) migrants needed to contribute to the SSS fund for at least six months prior to the unemployment condition; (ii) they needed to complete self-quarantine as a result of the closure of their workplace; and (iii) their prior employment must have been part of the list announced by the government that dictated the organisations they believed to have been affected by COVID-19. Once all criteria were met, migrant workers received the 50% subsidy of their daily wages, but only for 90 days after the initiation of their unemployment condition [33].

### 3.3. Quality of Health Screening in MOU Migrants

Some participants suggested that, under the MOU policy, there should be an effort to work with the migrants’ countries of origin in order to establish proactive health screening and surveillance programs in those settings before their arrival. This approach can improve control measures for infectious diseases, in particular HIV and syphilis, and mitigate transmission risk along the border of Thailand. However, in practice, efforts to strengthen the health check mechanism at the country of origin were not well-structured. As such, strong preventive measures, such as health screening and surveillance and treatment before cross-border travel, should be well-established in neighbouring countries. Although both public and private health facilities had a role in health examinations as part of the NV process, the national policy message about the validity of health examinations was unclear.

“*The prevalence of HIV and syphilis is more intense in cross-border migrants. It will be more problematic if they get infected from their countries. Also, it means that its transmissibility will be greater in Thailand. Additionally, surveillance system for these infectious diseases is critical and it should be more proactive.*”

—Health Professional PA-5

“*We have already had the health checks from migrant workers’ countries of origin but practically the implementation is not good enough although this is a condition under the MOU. Sometimes our MOL does not care about the health check results. They try to urge them to have work permits at first and then the health check.*”

—Health Professional PA-17

### 3.4. Health Screening Problems and State Quarantine Management in Response to COVID-19

The demand for importing MOU migrant workers to Thailand still continued during COVID-19. To prevent the spread of COVID-19, the MOL asked the private sector to be responsible for the cost of health screening and quarantine for imported migrants [34]. It was estimated that the cost of COVID-19 screening and quarantine was approximately THB 13,200–19,300 (USD 400–585) per person. However, it was not clear whether this cost would be subsidised by migrants themselves or by the employers.

Some interviewees suggested that the Social Security Office (the governing body of the SSS) or the DHEHS of the Thai MOPH (the governing body of the HICS) should take care of the cost for state quarantine. In the meantime, there was a contrasting view about whether it would be difficult for the HICS funding to be utilised for such costs. The reason being that this expense would affect the sustainability of the HICS fund in the future. Moreover, as the purpose of the HICS is specifically reserved for medical treatment, the cost of health screening and state quarantine for COVID-19 was unlikely to be valid for HICS fund utilisation [35]. One of respondents suggested that during this crisis, a key authority in the MOPH responsible for the cost of screening and state quarantine should be clearly identified.

“*There is a gap in the financial policy to identify who should be the key player responsible for the cost of COVID-19 screening. Currently, the Department of Disease Control is bearing this cost but some told us that the DHEHSF has a direct mission to take care of migrant people. However, the HICS reimbursement does not cover COVID-19. I am so worried about this situation as now the Department of Disease Control and some health facilities have to use their own finances for the screening during this crisis.*”

—Health Professional PA-17

One of the interviewees said he was not sure that a shorter quarantine period could be implemented to reduce the cost. It was estimated that the average cost of quarantine for one migrant worker was about THB 1500 (USD 50) [24]. However, at the time of the writing, the period for quarantine remained at 14 days. The decision to reduce the quarantine period was not yet finalised officially by the government.

“*It is unclear about the appropriate settings for the state quarantine. Some people suggested that migrants can be quarantined at the factories but we cannot ensure the quality of that. Now there is a negotiation to reduce the cost of state quarantine with a shorter period.*”

—Health Professional PA-5

### 3.5. Managing Migration Quota and Dependency on Migrant Workers

According to the regulations on MOU migrant workers, employers can request a quota to import migrant workers to Thailand. In this process, they need to identify the number of migrant employees and the information related to the type of work. However, some participants mentioned that at the macro level they did not realise the actual demand for migrant workers (especially the low-skilled ones). They also suggested that the Office of the National Economic and Social Development Council (NESDC) should be a national leader in developing this framework and set up a quota for importing migrants within a specific timeframe. This idea would help create a sound balance between labour demand and national security. At present, there is an effort to identify the ceiling for imported migrants in each business sector to prevent an excessive migrant import [36]. However, this needs a clear information system on the number of undocumented migrants from the outset.

“*The NESDC should speak out about how many low-skilled migrants should be imported from neighbouring countries within 5 years. This number needs to be identified, along with how many of them can boost the national economy.*”

—Health Professional PA-1

“*In fact we should accept that there are many undocumented migrants in Thailand. We need to know who they are, where they live, and how we can deal with them. The government needs to have a plan and policy to address this issue and report the accurate number of undocumented migrants. Big data system is absent in Thailand which is a way to report and track some personal information systematically such as their ID.*”

—International Organisation Officer PA-10

### 3.6. Influx of Migrants in the Backdrop of COVID-19

The social discourse during early 2021 claimed that the undocumented status of migrants was one of the main reasons for the second wave of COVID-19 in Thailand. A quick survey reported that the majority of Thai participants perceived that the superspreading event was caused by the irresponsibility of government authorities in dealing with undocumented migrants [37]. Some participants agreed that although the situation of COVID-19 in Thailand remained critical, this could be an opportunity to urge the government to expedite the NV process [37]. So far, the government has mitigated the illegality problem by just extending the visa for migrant workers from Myanmar, Cambodia, and Lao PRD to the end of 2021 [38].

“*COVID-19 is an opportunity to revise the regulation on undocumented migrants. Though this crisis remains in the country, we still need migrant workers. A long-term plan for addressing undocumented migrants should be developed, and it should be not only a 5-year plan but rather 10 years.*”

—International Organisation Officer PA-10

In addition, due to the protest against the military regime in Myanmar, there was a huge influx of migrant workers and refugees crossing the border to Thailand [39]. The Thai Government anticipated an influx of refugees due to the political unrest in Myanmar and had taken measures to mitigate the impact [40]. This situation in Myanmar also impeded the progress of the NV as this required mutual coordination between the country of origin (Myanmar) and the destination country (Thailand). Some participants raised a concern about the surge of COVID-19 imported cases as a result of the political conflict in Myanmar. One of respondents noted that the capacity of the Thai health system might not be sufficient to address the influx of undocumented migrants from Myanmar during the political unrest.

“*Opponents of the military regime will definitely come to Thailand. I am not sure how the local health authorities could deal with it. Actually, there should be temporary camps to help people fleeing from Myanmar but I am not sure whether now it is in place.*”

—Government Officer PA-15

### 3.7. Poor Living Condition of Migrants and the Impact of COVID-19

The second wave of COVID-19 in late 2020 originated in Samut Sakhon, a provincial vicinity of Bangkok and one of the most densely migrant-populated areas in the country. This situation caught the wider public’s attention, raising awareness about the poor working and cramped living conditions of migrants. One participant suggested that poor living conditions were a critical factor that facilitated disease transmission. The crowding in migrant communities caused significant difficulties in maintaining physical distancing, and a significant number of migrants were still in need of basic support during the COVID-19 crisis. A rapid assessment by the International Organisation for Migration (IOM) in Samut Sakhon found that about 12% of migrants needed food assistance, and the same proportion (12%) were unable to access basic healthcare [41].

“*Our concern is about their poor living conditions. Actually, food and housing are even worse than for Thais. The Ministry of Interior needs to take action to improve their quality of life. Employers have to take care of them. However, many employers, I have heard, also seek decent rooms for their migrant workers. Many of them are living in a small room, and the water quality is also poor.*”

—Health Professional PA-5

## 4. Discussion

Seven key themes emerged from the analysis through the inductive and deductive thematic coding. Regarding the themes of financial systems for migrants, the issue of the sustainability of the HICS funding was raised; integrating the HICS with the UCS was considered a policy option. Low utilisation of the SSS fund was found amongst migrant workers, especially for the additional benefits beyond medical treatment. In terms of access to health services, the quality of health screening and state quarantine was also points of contention. Although the government encouraged the private sector to take care of the quarantine expense in order to maintain the labour demand, the high cost of the process prohibited the involvement of the private sector. Although COVID-19 was perceived as the crisis, it could be seen as an opportunity to reorientate the Thai health system to be more inclusive for migrants regardless of their immigration status.

The healthcare policy for migrants is very complex as it is hugely influenced by the concept of national security and economic necessity [3]. Although Thailand has implemented two main health insurance schemes for migrant workers (the SSS and HICS) for over a decade, the implementation of such schemes is dynamic and always faces challenges. The sustainability of migrant health insurance was often mentioned in the dialogue among the study interviewees. Consideration for the sustainable HICS fund was mentioned, and this may be due to the fact that it is operated on a semi-voluntary basis, making the pool funding small. In 2021, the total number of SSS beneficiaries in the formal sector (including all nationalities) was reported at THB 11 million (USD 0.34 million), while the number of HICS beneficiaries was found to be ten times lower than SSS beneficiaries at only THB 0.9 million [42]. This concern was reflected as it could affect the long-term sustainability of the HICS.

Due to the structural mechanism of the HICS, the institutional capacity of the HICS is limited compared with the SSS because the MOPH was not set up to be the fund manager (or the insurer) from the outset. The direction of migrant policy is dynamic, especially for the categories of adult dependents [10]. As such, these factors impede success in developing tools for the administrative work of the HICS. Similar to previous studies, we found that both migrants and their employers are reluctant to contribute to the SSS due to the perceived unaffordability of the insurance [10,20]. However, these structural challenges are not worsened by the SSS and HICS themselves; the driver is the underlying structural problem that excluded both schemes from the Thai UCS [16,43]. Integration of migrant health insurance in the Thai UCS cannot be achieved with a short-term response; it takes time to identify political feasibility through the lens of health equity and an operational system.

For undocumented migrants, facilitating the process of the NV and MOU is recognised as the first step to deal with precarious status and accelerate the uptake of health insurance [20]. Such process should be improved by simplifying complexity in the registration system; making it more affordable; and reducing bureaucratic procedures [3,20]. A long-term plan for managing the labour demand is necessary for the government to be better prepared for resource allocation in response to the crisis [20].

Challenges in the number of undocumented and documented migrants and migrant health insurance are also pronounced in ASEAN countries, including Malaysia and Singapore, two of the most important destination countries in the region. In Malaysia, documented migrants account for 15% of the total domestic labour force [44,45]. All documented migrant workers, except those in the plantation sector and domestic workers, are covered by mandatory health insurance, the Foreign Worker Hospitalization and Insurance Scheme (SPIKPA) and social security, the Employment Injury Scheme for Foreign Workers under the government’s Social Security Organisation (EI-SOCSO). Although these health insurance and social security schemes cover expenses from hospital admissions and surgery at Ministry of Health, and hospitals and occupational illness or injury benefits, financial barriers to accessing healthcare services were reported as higher fees for noncitizens at public healthcare facilities were prohibitive [11,44]. Barriers to healthcare access included out-of-pocket payment for outpatient clinic visits, a lack of awareness about insurance provisions and eligibilities, inadequacy in covering costs of medical treatment, and institutional discrimination of migrant workers [11,44]. For undocumented migrants, the situation is even more problematic as they are neglected by the main public health policies [11,44,46]. A major barrier to utilisation is the need to present legal documents such as passports and work permits at healthcare facilities, putting undocumented migrant workers at risk of detention and deportation. Moreover, it was found that undocumented migrant workers tended to avoid hospital care unless they were either unconscious or critically ill, resulting in poor outcomes and avoidable deaths [11]. In 2016, an innovative Refugee Medical Insurance (REMEDI) was launched by the United Nations High Commissioner for Refugees (UNHCR), Malaysia, in partnership with RHB Insurance [47]. However, the scheme did not have long-term success and was suspended due to an increase in claim rates with low enrollment [44]. In Singapore, migrant workers have become a significant part of the labour market [48]. It was estimated that about 37% of the national work force were migrant workers [49]. The largest group with work permits were from Bangladesh, India, and China [48,49]. Health insurance for migrant workers in Singapore was separated from UHC for the Singaporeans. Healthcare financing for migrant workers was based on ‘employer responsibility’ under mandatory private medical insurance and private work injury compensation insurance [48]. However, challenges in access to healthcare were less described with no official statistical reports [48]. A recent review by Rajaraman et al. [48] ascribed the problems related to inaccessibility of care among migrants in Singapore to high costs of healthcare, gatekeeping among the employers, and vulnerability to repatriation among documented migrants. Ang et al. [50] conducted a cross-sectional study and found that low-wage migrant workers faced high costs in outpatient care. Moreover, catastrophic illness could not be fully covered by the insurance [50]. Additionally, the lack of knowledge on the coverage eligibility of health insurance and cultural and language barriers presented major gaps in healthcare access [51].

It is undeniable that COVID-19 massively impacts the health and wellbeing of populations with higher vulnerabilities. Migrant workers are disproportionately affected by COVID-19. Challenges were more pronounced, in particular inaccessibility to health and social services and crowded living conditions. During this crisis situation, strengthening the inclusiveness of public health efforts is key to containing and mitigating the outbreak and accelerating recovery across all populations [52]. During the first wave of COVID-19 in Thailand, the influx of new migrant workers was not permitted and the government launched a policy for migrant visa extension to sustain the domestic labour force [38]. Nevertheless, owing to the high expenditure for lawfully importing migrant workers, challenges with undocumented migrants cannot be solved immediately. This problem also critically impedes active screening in local areas and creates a situation where migrants may act as superspreaders of COVID-19 if they are excluded from the formal quarantine system. Additionally, the organisations responsible for financing the cost of state quarantine and vaccination for the migrant populations need to be clearly defined to determine whether the financial responsibility lies solely with the government or if it is covered through a partnership between the government and the employers (private sector). More importantly, this is the opportune time for reforming the NV and to attract the documentation of more undocumented migrants through strong collaboration between the government departments and the private sector. The dialogue between the Thai government and migrants’ countries of origin should be strengthened during this time of crisis to better address the structural problems that affect cross-border movement. These may include medical supply sharing and referral systems, knowledge transfer, and information systems for cross-border travel tracking. Moreover, migrant health workers, volunteers, and NGOs can play a vital role in promoting health literacy and transferring knowledge to the target migrants. However, marginalised social groups, including migrant workers, refugees, and asylum seekers, are particularly vulnerable and are affected by broader health impacts [53]. Horizontal and people-centred approaches with decentralisation and community-based programs tend to be effective in building the capacity of migrant communities to fight against the pandemic or at least survive during the COVID-19 era [53,54]. All of these accounts underpin the importance of holistic policy approaches that incorporate views and concerns from all stakeholders (the government that upholds national security, the business sector that aims to maintain its business, the NGOs and the civic groups, and also the migrant communities as the direct beneficiary) to address an extremely complex issue such as migrant health, and the even more complex challenge of migrant health during the global pandemic.

The COVID-19 crisis in the ASEAN countries is critical, and its impact is evident among migrant workers. In Singapore, this pandemic has widened health disparities in comparison with general populations [55]. Many migrants in Singapore have faced poor living environments, difficulties in social distancing, and healthcare discrimination including limited access to health information [50,55]. Yi et al. [56] highlighted structural changes with regard to poor legal and social protection to contain COVID-19 with a much-needed impact on the adequate living conditions of low-wage migrant workers in Singapore. At the national level, a whole-of-government approach, the Multi-Ministry Task Force, was established in Singapore as an implementing body for health measures to contain COVID-19 [57,58,59]. The activities included active case finding, contract tracing, state quarantine, clinical medicine, and social and community interventions to curb the crisis [56,58]. However, the regular monitoring system of these activities did not always function well [56,59].

Although the purpose of this study was to gather opinions from stakeholders in the field of migrant health policy, ranging from local actors to policymakers, some limitations remain. First, as there were difficulties due to travel restrictions during the study period, the information from the demand side, including from the migrant workers and their employers, was absent. However, interviews of representatives of the NGO organisations, who are key informants, were performed. Although these persons may not directly reflect the view of migrants themselves, only this option was available during the time of travel restriction, and they were able to share their insights about their extensive experience in working with migrants in the local context. Second, due to the changing COVID-19 situation and the dynamics of the policy response, this analysis captured only a snapshot of interviewees’ views, while the impact of COVID-19 on migrants’ health and wellbeing should be regularly monitored, including long-term consequences. Third, our analysis was mainly based on the public health lens and therefore insights into social and political dimensions as a whole were not comprehensively analysed in the findings. Rather, the challenges in healthcare faced by migrant workers were central to the analysis. It would be of great value to rethink the inclusion of the HICS in the Thai UHC with more comprehensive policies for COVID-19 and emerging diseases. Moreover, studies on the revision of MOUs for a labour force between Thailand and migrants’ country of origin and COVID-19 need to be considered with a broader scope including economic necessity and national security.

## 5. Conclusions

Multidimensional effects of COVID-19 have been confirmed worldwide. In this study, all participants agreed that COVID-19 affected migrant policies across the country. Due to a rapid increase in the confirmed cases, infection among undocumented migrants is of great concern, and the numbers tend to be underestimated. Regarding policy implications, in the short term, housing measures to avoid the overcrowded and unhygienic living conditions of migrant workers need to be addressed. In the long run, inclusiveness in social protection and public health policies needs to be enhanced to cover all vulnerable populations regardless of their nationality or status. A long-term plan at the national level is required for tackling the undocumented status of migrants, rather than just treating infected migrants as part of the immediate response during the crisis. Multisectoral coordination and collective actions within government agencies and between the government and other non-state actors, including civic groups, the business sector, and migrant communities themselves, are crucial. To improve implications of these findings, further studies should engage more stakeholders beyond the health sector and migrants themselves. This will provide deeper insights into the link between health and social policies for migrant communities, especially during the global crisis.

## Figures and Tables

**Table 1:** Characteristics of participants included in the study.

Code	Gender	Experience (Years)	Career Level	Organization
PA-1	Male	>10	Senior	Health sector (beyond the government)
PA-2	Male	>10	Middle	NGOs ^1^ (migrant working group)
PA-3	Female	5–10	Middle	MOPH ^2^
PA-4	Male	>10	Senior	MOPH ^2^
PA-5	Male	>10	Senior	MOPH ^2^
PA-6	Male	>10	Senior	Government
PA-7	Male	>10	Middle	NGOs ^1^ (migrant working group)
PA-8	Female	>10	Senior	NGOs ^1^ (Thai social foundation)
PA-9	Male	>10	Senior	Government
PA-10	Female	>10	Middle	NGOs ^1^ (international)
PA-11	Female	>10	Middle	NGOs ^1^ (international)
PA-12	Female	>10	Senior	NGOs ^1^ (international)
PA-13	Female	5–10	Middle	NGOs ^1^ (migrant working group)
PA-14	Female	<5	Middle	MOPH ^2^
PA-15	Female	>10	Senior	Health sector (beyond the government)
PA-16	Female	>10	Middle	Academic
PA-17	Male	5–10	Senior	MOPH ^2^
PA-18	Male	>10	Senior	Academic

^1^ NGOs: non-governmental organisations; ^2^ MOPH: the Ministry of Public Health of Thailand.

## Data Availability

Data available on request due to ethical restrictions.

## Abbreviations

ASEAN	Southeast Asia Nations
CLM	Cambodia, Lao PDR, and Myanmar
OSS	One Stop Service
MOPH	Ministry of Public Health of Thailand
MOI	Ministry of Interior
MOL	Ministry of Labor
MOU	Memorandum of Understanding
NV	Nationality Verification
UHC	Universal Health Coverage
UCS	Universal Coverage Scheme
CSMBS	Civil Servant Benefit Scheme
SSS	Social Security Scheme
HICS	Health Insurance Card Scheme
DHEHS	Division of Health Economics and Health Security
COI	Conflict of Interest
NESDC	National Economic and Social Development Council

## References

[1] Guinto R.L.L.R., Curran U.Z., Suphanchaimat R., Pocock N.S. (2015). Universal health coverage in One ASEAN: Are migrants included?. Glob. Health Action.

[2] ASEAN (2008). ASEAN Economic Community Blueprint.

[3] Suphanchaimat R., Putthasri W., Prakongsai P., Tangcharoensathien V. (2017). Evolution and complexity of government policies to protect the health of undocumented/illegal migrants in Thailand—The unsolved challenges. Risk Manag. Healthc. Policy.

[4] Lee E.S. (1966). A Theory of Migration. Demography.

[5] De Haas H. (2021). A theory of migration: The aspirations-capabilities framework. Comput. Migr. Stud..

[6] Castles S. (1998). The Age of Migration: International Population Movements in the Modern World.

[7] Paitoonpong S., Chalamwong Y. (2012). Managing International Labor Migration in ASEAN: A Case of Thailand.

[8] Piore M.J. (1979). Birds of Passage: Migrant Labor and Industrial Societies.

[9] Pasadilla G.O. (2011). Social Security and Labor Migration in ASEAN.

[10] Harkins B. (2019). Thailand Migration Report 2019.

[11] Loganathan T., Rui D., Ng C.-W., Pocock N.S. (2019). Breaking down the barriers: Understanding migrant workers’ access to healthcare in Malaysia. PLoS ONE.

[12] Orcutt M., Patel P., Burns R., Hiam L., Aldridge R., Devakumar D., Kumar B., Spiegel G., Abubakar I. (2020). Global call to action for inclusion of migrants and refugees in the COVID-19 response. Lancet.

[13] Alahmad B., Kurdi H., Colonna K., Gasana J., Agnew J., Fox M.A. (2020). COVID-19 stressors on migrant workers in Kuwait: Cumulative risk considerations. BMJ Glob. Health.

[14] Koh D. (2020). Migrant workers and COVID-19. Occup. Environ. Med..

[15] Greenaway C., Hargreaves S., Barkati S., Coyle C.M., Gobbi F., Veizis A., Douglas P. (2020). COVID-19: Exposing and addressing health disparities among ethnic minorities and migrants. J. Travel Med..

[16] Suphanchaimat R., Pudpong N., Prakongsai P., Putthasri W., Hanefeld J., Mills A. (2019). The Devil Is in the Detail-Understanding Divergence between Intention and Implementation of Health Policy for Undocumented Migrants in Thailand. Int. J. Environ. Res. Public Health.

[17] WHO South-East Asia Region (2018). Health of Refugees and Migrants: Parctices in Addressing the Health Needs of Refugees and Migrants. https://www.who.int/migrants/publications/PAHO-Practices.pdf.

[18] Posttoday, A Request from Illegal Migrant Workers for Nationality Verification. https://www.posttoday.com/social/general/645343.

[19] Division of Health Economics and Health Security Notification of the Ministry of Public Health of Thailand Re: Health Examination and Health Insurance for Migrant Workers 2019. https://dhes.moph.go.th/?p=4869.

[20] Kunpeuk W., Teekasap P., Kosiyaporn H., Julchoo S., Phaiyarom M., Sinam P., Pudpong N., Suphanchaimat R. (2020). Understanding the Problem of Access to Public Health Insurance Schemes among Cross-Border Migrants in Thailand through Systems Thinking. Int. J. Environ. Res. Public Health.

[21] Tangcharoensathien V., Patcharanarumol W. (2019). The Political Economy of UHC Reform in Thailand: Lessons for Low- and Middle-Income Countries. Health Syst. Reform.

[22] Department of Employment. Ministry of Labour of Thailand Guidelines for Documented Migrant Workers in Thailand. https://bit.ly/2WkFkFC.

[23] Bangkokpost Don’t Leave Migrant Workers Behind. https://www.bangkokpost.com/business/2154731/dont-leave-migrant-workers-behind.

[24] Thailand Development Research Institute (2020). The Impact of COVID-19 on the Mangement of Migrant Workers in Thailand. https://tdri.or.th/2020/08/covid-19-impact-on-migrant-workers/.

[25] National News Bureau of Thailand Infectious and High-Risk Areas in Greater Bangkok Will Be Closed Off. https://thainews.prd.go.th/en/news/detail/TCATG210626111504398.

[26] Thairath Cancellation on Active Case Finding in Documented Migrant Workers: Failure of the Thai Government with Ignorance of Migrant Workers in the Country. https://www.thairath.co.th/scoop/theissue/2141952.

[27] Abubakar I., Aldridge R.W., Devakumar D., Orcutt M., Burns R., Barreto M.L., Dhavan P., Fouad J.M., Groce M., Guo Y. (2018). The UCL–Lancet Commission on Migration and Health: The health of a world on the move. Lancet.

[28] Levesque J.-F., Harris M.F., Russell G. (2013). Patient-centred access to health care: Conceptualising access at the interface of health systems and populations. Int. J. Equity Health.

[29] OHCHR Principles and Guidelines, Supported by Practical Guidance, on the Human Rights Protection of Migrants in Vulnerable Situations Reports A/HRC/37/34 9. https://www.ohchr.org/Documents/Issues/Migration/OHCHR_slow_onset_of_Climate_Change_ENweb.pdf.

[30] Poungkanta W., Suphanchaimat R. (2017). Health insurance for undocumented migrants: A literature review in developed countries. J. Med. Assoc. Thail..

[31] Witthayapipopsakul W., Kulthanmanusorn A., Vongmongkol V., Viriyathorn S., Wanwong Y., Tangcharoensathien V. (2019). Achieving the targets for universal health coverage: How is Thailand monitoring progress?. WHO South-East Asia J. Public Health..

[32] Prachachat, The Social Security Scheme for Migrant Workers in Thailand is the Same as the Thais. https://www.prachachat.net/csr-hr/news-325611.

[33] Ministry of Labor of Thailand (2021). New Normal during the COVID-19 and Labor of Thailand: Get through the COVID-19 Together. E-Mag. Min. Labor Thai. Bang..

[34] Thai Civil Rights and Investigate Journalism Migrant Employers Asked for Subsidies on Health Screeing and State Quarantine before Importing Migrant Workers in Thailand. https://www.tcijthai.com/news/2020/7/scoop/10722.

[35] The Ministry of Public Health of Thailand Minute of the Meeting on Healthcare Intervention and Services for Migrants, Migrant Workers, Refugees, and Stateless Persons in Thailand during COVID-19 as of 29 April 2020. http://www.cbo.moph.go.th:8080/welfare/document_files/20200526-1590474881.pdf.

[36] Puey Ungphakorn Institute for Economic Research (2020). Facts about Migrant Workers in Thailand: Chapter 1 Low-Skilled Workers through the Lens of the Social Security Fund. https://www.pier.or.th/abridged/2020/14/.

[37] Hfocus (2021). The Results of the Public Poll regarding Ilegal Migration and COVID-19. https://www.hfocus.org/content/2021/02/21049.

[38] Ministry of Interior of Thailand Notification of the Ministry of Interior of Thailand Re: Permission on Visa Extension for Specific Grooups of Migrant Workers in Thailand during the Situation of COVID-19. http://www.ratchakitcha.soc.go.th/DATA/PDF/2563/E/305/T_0012.PDF.

[39] Bangkokpost Myanmar Protests Pose Virus Threat. https://www.bangkokpost.com/thailand/general/2072171/myanmar-protests-pose-virus-threat.

[40] DW News Thousands Flee Military to Thailand-Myanmar Border Region. https://www.dw.com/en/thousands-flee-military-to-thailand-myanmar-border-region/a-57007896.

[41] International Organization for Migration (2021). COVID-19 Rapid Needs Assessment: Mahachai Sub-District Samut Sakhon Province (Round 4). https://reliefweb.int/sites/reliefweb.int/files/resources/R4%20Needs%20Assessment_Mahachai_0.pdf.

[42] Ministry of Public Health of Thailand Data on the HICS Registry with Valid Status. https://data.go.th/tr/dataset/dhesmoph.

[43] Herberholz C. (2020). The Role of External Actors in Shaping Migrant Health Insurance in Thailand. PLoS ONE.

[44] Loganathan T., Chan Z.X., Pocock N.S. (2020). Healthcare financing and social protection policies for migrant workers in Malaysia. PLoS ONE.

[45] Department of Statistics Malaysia (2018). Key Statistics of Labour Force in Malaysia, July 2018.

[46] Hacker K., Anies M.E., Folb B., Zallman L. (2015). Barriers to health care for undocumented immigrants: A literature review. Risk Manag. Health Policy.

[47] Chuah F.L.H., Tan S.T., Yeo J., Legido-Quigley H. (2019). Health System Responses to the Health Needs of Refugees and Asylum-seekers in Malaysia: A Qualitative Study. Int. J. Environ. Res. Public Health.

[48] Rajaraman N., Yip T.-W., Kuan B.Y.H., Lim J.F.Y. (2020). Exclusion of Migrant Workers from National UHC Systems—Perspectives from HealthServe, a Non-profit Organisation in Singapore. Asian Bioeth. Rev..

[49] Ministry of Manpower (2019). Foreign Workers Continue to Rate Working in Singapore Favourably in Latest Survey: Pay, Living Conditions, Safety and Security Commonly Cited Reasons. https://www.mom.gov.sg/newsroom/press-releases/2019/0609-foreign-workers-continue-to-rate-working-in-singaporefavourably-in-latest-survey.

[50] Ang J.W., Chia C., Koh C.J., Chua B.W.B., Narayanaswamy S., Wijaya L., Chan L.G., Goh W.L., Vasoo S. (2017). Healthcare-seeking behaviour, barriers and mental health of non-domestic migrant workers in Singapore. BMJ Glob. Health.

[51] Ang J., Koh C., Chua B., Narayanaswamy S., Wijaya L., Chan L., Soh L., Goh W., Vasoo S. (2020). Are migrant workers in Singapore receiving adequate healthcare? A survey of doctors working in public tertiary healthcare institutions. Singap. Med. J..

[52] Berger Z.D., Evans N.G., Phelan A., Silverman R.D. (2020). COVID-19: Control measures must be equitable and inclusive. BMJ.

[53] Hrynick T.A., Lorenzo S.R., Carter S.E. (2021). COVID-19 response: Mitigating negative impacts on other areas of health. BMJ Glob. Health.

[54] WHO (2020). Neglected Tropical Diseases: Leveraging the New NTD Road Map to Build Back from COVID-19 Disruptions. https://www.who.int/news/item/19-06-2020-neglected-tropical-diseases-leveraging-the-new-ntd-road-map-to-build-back-from-covid-19-disruptions.

[55] Goh O.Q., Islam A.M., Lim J.C., Chow W.-C. (2020). Towards health market systems changes for migrant workers based on the COVID-19 experience in Singapore. BMJ Glob. Health.

[56] Yi H., Ng S.T., Farwin A., Low A.P.T., Chang C.M., Lim J. (2020). Health equity considerations in COVID-19: Geospatial network analysis of the COVID-19 outbreak in the migrant population in Singapore. J. Travel Med..

[57] Chiew C.J., Li Z., Lee V.J. (2020). Reducing onward spread of COVID-19 from imported cases: Quarantine and stay at home measures for travellers and returning residents to Singapore. J. Travel Med..

[58] Archuleta S., Cross G., Somani J., Lum L., Santosa A., Alagha R.A., Allen D.M., Ang A., Beh D., Chai L. (2020). Responding to COVID-19: How an academic infectious diseases division mobilized in Singapore. BMC Med..

[59] Lee V.J., Chiew C.J., Khong W.X. (2020). Interrupting transmission of COVID-19: Lessons from containment efforts in Singapore. J. Travel Med..

